# Linoleic acid pathway disturbance contributing to potential cancerization of intrahepatic bile duct stones into intrahepatic cholangiocarcinoma

**DOI:** 10.1186/s12876-022-02354-2

**Published:** 2022-05-30

**Authors:** Jun Li, Jiongjiong Lu, Shaodong Lv, Shujun Sun, Caifeng Liu, Feng Xu, Haiying Sun, Jiamei Yang, Xinjun Wang, Xingyang Zhong, Junhua Lu

**Affiliations:** 1 The 1st Department of Hepatic Surgery, Eastern Hepatobiliary Surgery Hospital, Second Military Medical University, Shanghai, China; 2 Department of Special Treatment, Eastern Hepatobiliary Surgery Hospital, Second Military Medical University, Shanghai, China; 3 The 5st Department of Hepatic Surgery, Eastern Hepatobiliary Surgery Hospital, Second Military Medical University, Shanghai, China; 4 School of Biology and Food Engineering, Fuyang Normal University, Fuyang, China; 5 Institute of Intestinal Diseases, Shanghai Tenth People’s Hospital, School of Medicine, Tongji University, Shanghai, China; 6 Shanghai Institution of Gut Microbiota Research and Engineering Development, Tenth People’s Hospital of Tongji University, Tongji University School of Medicine, Shanghai, China

**Keywords:** Intrahepatic cholangiocarcinoma, Intrahepatic bile duct stone, Linoleic acid, Metabolomics, GC–MS

## Abstract

**Background:**

Intrahepatic cholangiocarcinoma (ICC) is the second most common primary hepatic malignancy with poor prognosis. Intrahepatic bile duct stone (IBDS) is one of the key causes to ICC occurrence and can increase morbidity rate of ICC about forty times. However, the specific carcinogenesis of IBDS is still far from clarified. Insight into the metabolic phenotype difference between IBDS and ICC can provide potential mechanisms and therapeutic targets, which is expected to inhibit the carcinogenesis of IBDS and improve the prognosis of ICC.

**Methods:**

A total of 34 participants including 25 ICC patients and 9 IBDS patients were recruited. Baseline information inclusive of liver function indicators, tumor biomarkers, surgery condition and constitution parameters etc. from patients were recorded. ICC and IBDS pathological tissues, as well as ICC para-carcinoma tissues, were collected for GC–MS based metabolomics experiments. Multivariate analysis was performed to find differentially expressed metabolites and differentially enriched metabolic pathways. Spearman correlation analysis was then used to construct correlation network between key metabolite and baseline information of patients.

**Results:**

The IBDS tissue and para-carcinoma tissue have blurred metabolic phenotypic differences, but both of them essentially distinguished from carcinoma tissue of ICC. Metabolic differences between IBDS and ICC were enriched in linoleic acid metabolism pathway, and the level of 9,12-octadecadienoic acid in IBDS tissues was almost two times higher than in ICC pathological tissues. The correlation between 9,12-octadecadienoic acid level and baseline information of patients demonstrated that 9,12-octadecadienoic acid level in pathological tissue was negative correlation with gamma-glutamyl transpeptidase (GGT) and alkaline phosphatase (ALP) level in peripheral blood. These two indicators were all cancerization marker for hepatic carcinoma and disease characteristic of IBDS.

**Conclusion:**

Long-term monitoring of metabolites from linoleic acid metabolism pathway and protein indicators of liver function in IBDS patients has important guiding significance for the monitoring of IBDS carcinogenesis. Meanwhile, further insight into the causal relationship between linoleic acid pathway disturbance and changes in liver function can provide important therapeutic targets for both IBDS and ICC.

**Supplementary Information:**

The online version contains supplementary material available at 10.1186/s12876-022-02354-2.

Cholangiocarcinoma is inclusive of intrahepatic cholangiocarcinoma (ICC), perihilar cholangiocarcinoma (PCC), and distal cholangiocarcinoma (DCC) according to the tumor location along the bile duct tree [1]. Among them, ICC as a malignant tumor originates from bile duct epithelium cells lining the intrahepatic ducts located proximal to the second degree of bile duct [2], and it is regarded as a typical subtype of primary liver cancer (PLC). The incidence of ICC has been reported to have been increasing worldwide over the past decades and the mortality of it is expected to reach nearly 50% by 2035 [3]. So far surgical resection is the only treatment option for curing this disease [4], even though liver transplantation might be effective for the patients with early ICC [5]. Even with surgery, the 5-year survival rate of patients is lower than 30%, and median survival is 27–36 months [4, 6]. The prognosis of this malignancy is dismal owing to its high propensity for regional and distant metastases [7]. The multifactorial etiologies and complicated environmental factors, which predispose to ICC, including chemical carcinogens, liver fluke infection, Hepatitis B Virus (HBV), hepatitis C virus (HCV), primary sclerosing cholangitis (PSC), hepatolithiasis (or intrahepatic bile duct stones, IBDS) [8], determine the inter-tumor genomic heterogeneity of ICC [9]. Some studies indicate that IBDS is an independent risk factor for ICC, and the total incidence of ICC caused by IBDS is 5%-13% in Asian populations [10, 11]. Notably, patients with IBDS-associated ICC have worse outcomes than patients with non-IBDS-related ICC [12]. Regrettably, the mechanism of evolution from IBDS into ICC is still not fully understood.

In recent years, as the end-point of omics technology, metabolomics technology has shown great advantage in clarifying pathogenesis of critical illness [13, 14]. Based on metabolomics research, series of biomarkers, including deoxycholic acid [15] and glycocholic acid [16] have been developed for hepatocellular carcinoma (HCC) diagnosis. Most recently, using untargeted metabolomics approach, Zhang et al. demonstrated metformin promoted glycolysis of cholangiocarcinoma cell and increased levels of BCAAs and UDP-GlcNAc, contributing to occurrence of autophagy and cell cycle arrest, and eventually present the anti-cancer role [17]. Tomacha et al. demonstrated cholangiocarcinoma-associated lipid biosynthesis disorder is related purine metabolism process, inclusive of the increase of guanine and glutamine level, which can be develop into potential biomarker for cholangiocarcinoma diagnosis. Murakami et al. further confirmed that ICC was associated with lipid metabolism and bile juice secretion, which inclusive of taurine, amino acid class and G-3-P, and eventually contributed to the metabolic reprogramming in ICC [18]. However, to the best of our knowledge, little attention has been focused on the correlation between IBDS and ICC in metabolomics study until recent years.

Herein, we performed GC–MS based metabolomics study using pathological tissue samples from 25 ICC patients and 9 IBDS patients (Scheme 1). Baseline information of liver function indicators, tumor biomarkers, surgery condition and constitution parameters et al. from patients were recorded for metabolite-phenotype based correlation analysis. Finally, the potential cancerization mechanism and diagnosis biomarkers of IBDS was expounded for monitoring and treatment application.

**Scheme 1:**
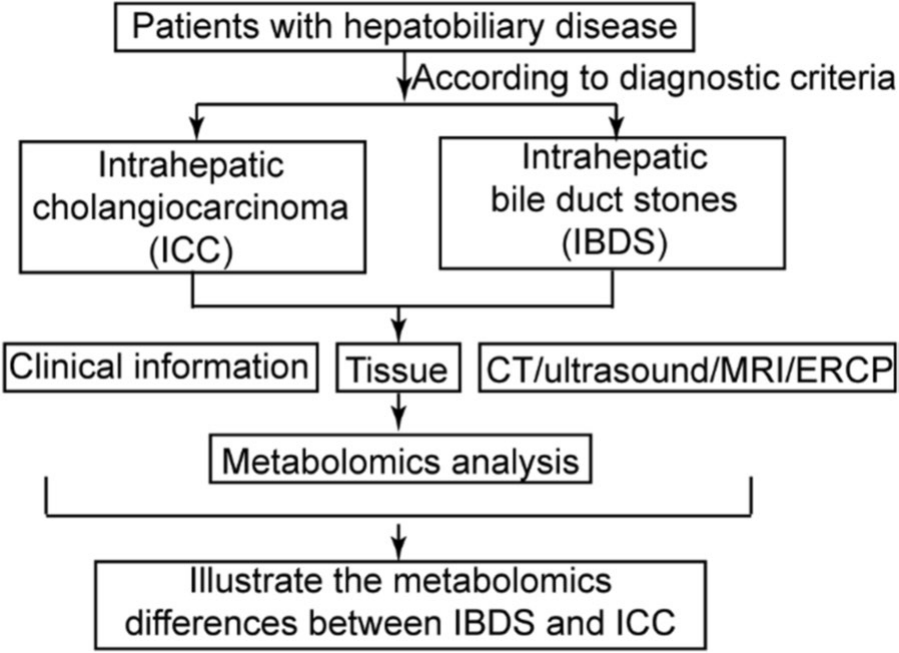
Flow diagram of the study

## Methods and materials

### Population, information, and sample collection

The clinical trial evaluated patients in hepatic surgery department of Eastern Hepatobiliary Surgery Hospital Affiliated to the Second military medical university from May 2016 to May 2017. Scheme 1 provides an overview of the study. A total of 33 subjects were recruited, 24 in the ICC group and 9 in IBDS group. All subjects provided written informed consent. General information was recorded at each subject’s first visit, including age, gender, and medical history et al. The results of laboratory tests (liver function and tumor markers), ultrasound and computed tomography (computed tomography, CT) were also recorded before surgery. Tissues for metabolic profiling were collected from surgery. More specifically, ICC tumor resection were performed centering on the tumor tissue during the operation; para-cancerous tissue is identified as the tissue 2 cm outside the tumor boundary. For patients with intrahepatic bile duct stones, anatomical hepatectomy is performed; the sampling location is the hepatic segment or hepatic lobe bile duct at the site of the calculus lesion, with a size of about 2 cm, and the area of bile duct thickening, sclerosis, or atrophy is usually the key sampling area. All tumor tissue and bile duct lesions must ultimately be confirmed by pathologists for histopathological diagnosis. The tissue specimens used in this study were all diagnosed by 3 pathologists, then frozen and used for subsequent GC–MS experimental detection.

### Diagnostic criteria

IBDS diagnosis was made by ultrasonography, computed tomography (CT), magnetic resonance imaging (MRI), or endoscopic retrograde cholangiopancreatography (ERCP). The diagnosis of ICC was established on pathology of biopsy or operative samples [16].

### Inclusion criteria

After diagnosis of IBDS and ICC, men and women aged 18–60 years with no history of treatment for liver disease were eligible. All patients signed the informed consent form for this study. This study was approved by the human research ethics committees of Eastern Hepatobiliary Surgery Hospital Affiliated to the Second Military Medical University.

### Exclusion criteria

Patients were excluded if they had any of the following: (1) history of metabolic diseases, including diabetes, alcoholic liver disease, non-alcoholic fatty liver disease, dyslipidemia, and unexplained abnormal liver function; (2) alcohol consumed > 20 g per day; (3) acute diseases or other untreated illness; (4) impaired heart or renal functions; (5) childbearing aged female who were pregnant or lactating; (6) prior receiving medication or other treatment; or (7) other conditions that may endanger subject safety or affect the study results.

### Sample collection and gas chromatography-mass spectrometry (GC–MS) analysis

Tissue samples were collected from each subject during biopsy or surgery and were frozen at − 80 °C until analysis. Details of GC–MS analysis were list in Additional file 1.

## Results

### Characteristics of study participants

**Fig. 1:**
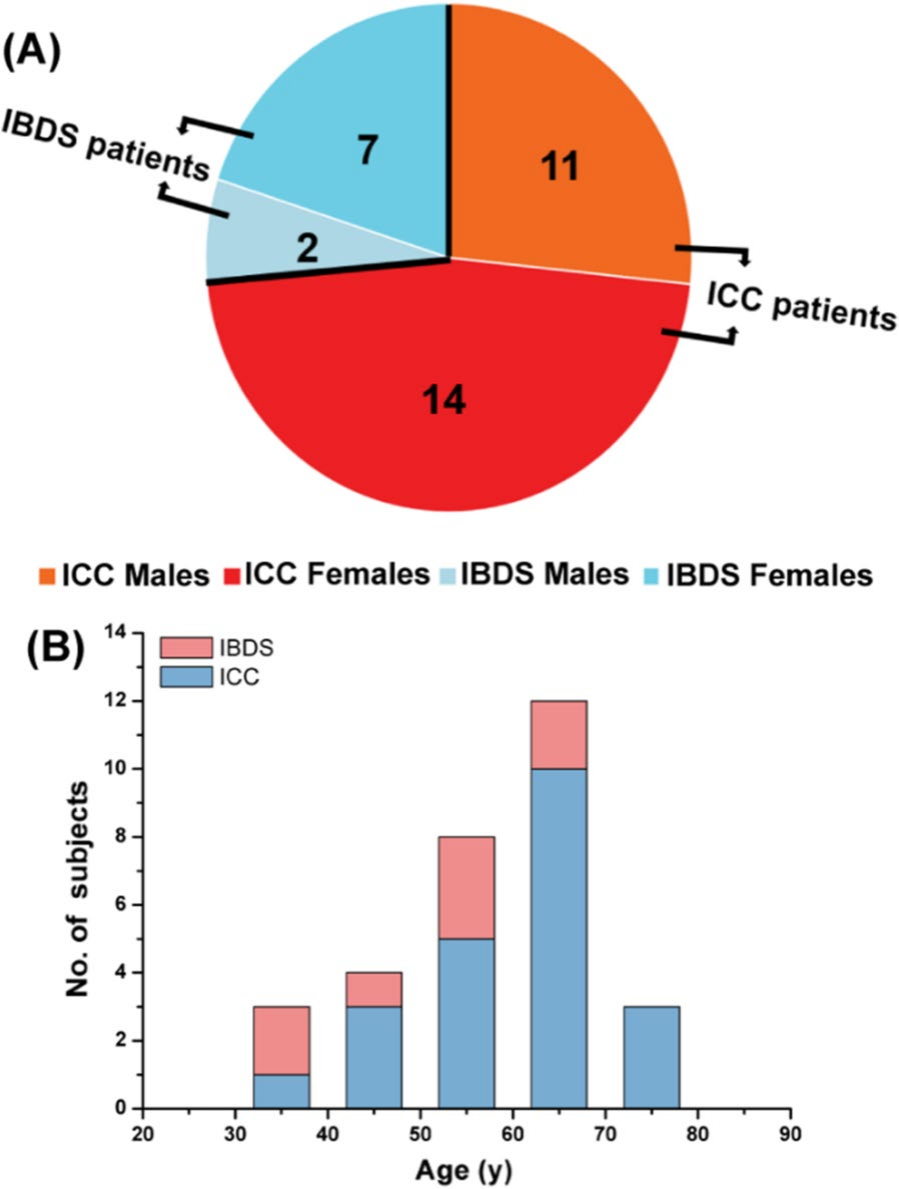
Overview of cohort characteristics in gender (**A**) and age (**B**)

To elucidate the possible molecular mechanism of IBDS evolved into ICC, we recruited 34 participants including 25 ICC patients and 9 IBDS patients. Pathological results are the basis of the diagnosis of these two diseases. The gender and age distribution in these two groups were presented in Fig. 1A, B. Detailed information of these patients, including baseline information, biochemical test results, tumor biomarker level, operation conditions and pathological diagnosis, was collected to demonstrate the possible relationship between ICC and IBDS. However, we found that without the assistance of imaging information, the above collected information can hardly be used to distinguish IBDS from ICC (Additional file 1: Fig. S1), so it could not provide effective clues for explaining how IBDS developing into ICC. Consequently, GC–MS-based metabolomics analysis was next performed by using tumor tissue, para-carcinoma tissue and IBDS pathological tissue as experimental object, hoping to find potential evidence on the metabolic level.

### Phenotype classification via GC–MS based metabolic fingerprinting

**Fig. 2:**
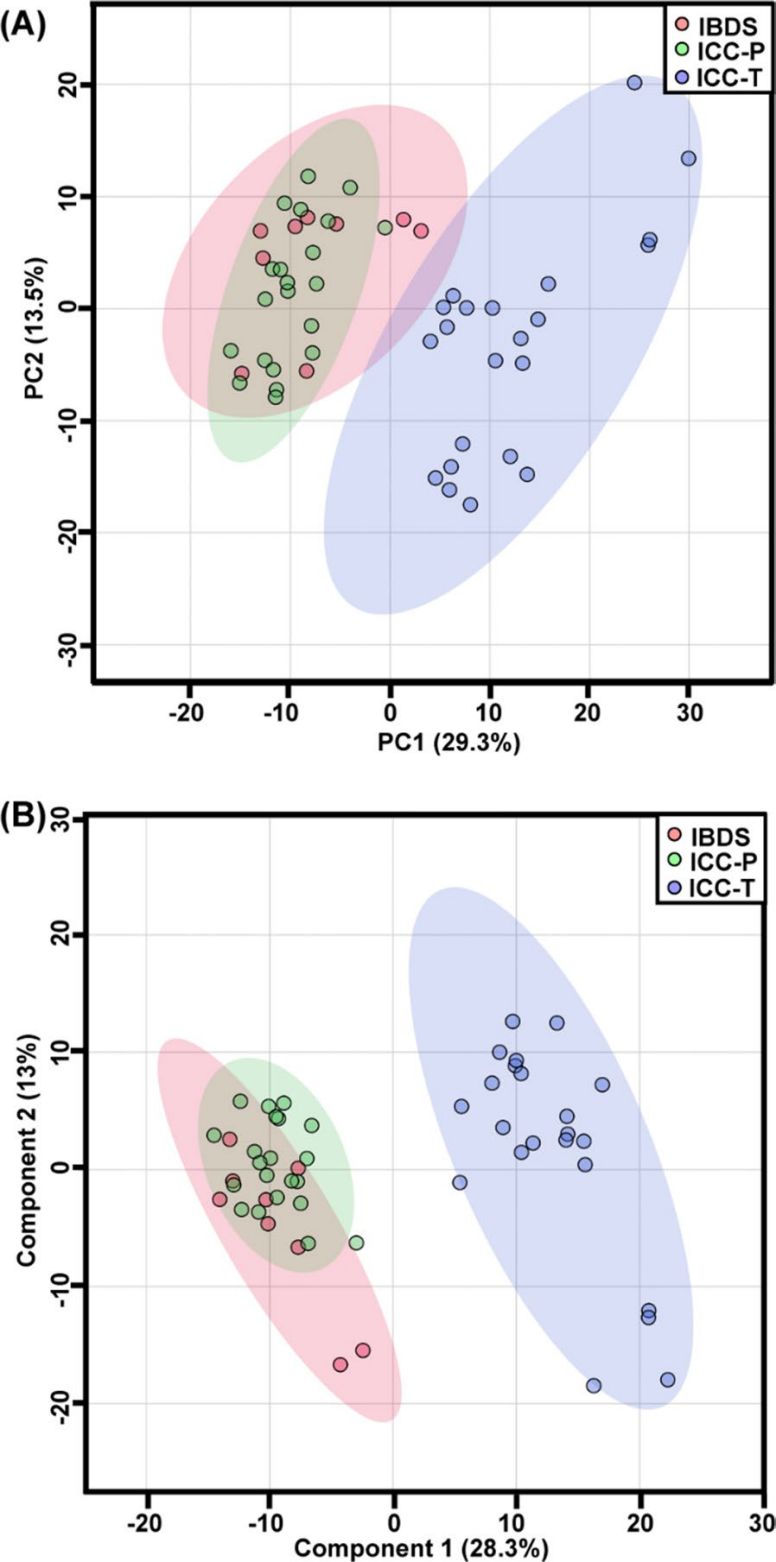
Classification of ICC carcinoma, para-carcinoma and IBDS via multivariate analysis. **A** S-plots following PCA analysis of three groups; **B** S-plots following PLS-DA analysis of three groups

Based on the metabolic fingerprinting obtained from GC–MS testing, we attempted to distinguish metabolic phenotypes of ICC tissues (abbr. as ICC-T) from non-cancerous counterparts including para-carcinoma tissues (abbr. as ICC-P) and IBDS. Through principal component analysis (abbr. as PCA), three groups of samples were separated into two group in the 2D scores plot (Fig. 2A). The metabolic phenotypes of ICC-T samples basically can be differentiated from ICC-P and IBDS samples with very little overlap of 95% confidence intervals. However, there was high similarity existed between ICC-P and IBDS. This similarity of IBDS and ICC-P may be attributed to their common cancerization trend, because 2–10% of Asian IBDS patients will eventually develop to ICC [19], significantly higher than normal Asian ICC incidence which was about 0.5–1.0‰ [8, 19–21]. Partial least squares-discriminant analysis (abbr. as PLS-DA) was further used to demonstrate the potential metabolic difference between IBDS and ICC. This PLS-DA model also used two principal components, with R^2^X of 78.4% and Q^2^ of 57.4%, indicating a great interpretability and predictability. But, the metabolic phenotype between IBDS and ICC-P still could not be distinguished in PLS-DA score plot (Fig. 2B). Therefore, we next tried to respectively conduct PLS-DA analysis with ICC-T-IBDS and ICC-T-ICC-P comparison to find their potential differential metabolic difference.

### Metabolic fingerprints difference between ICC-T and ICC-P

**Fig. 3:**
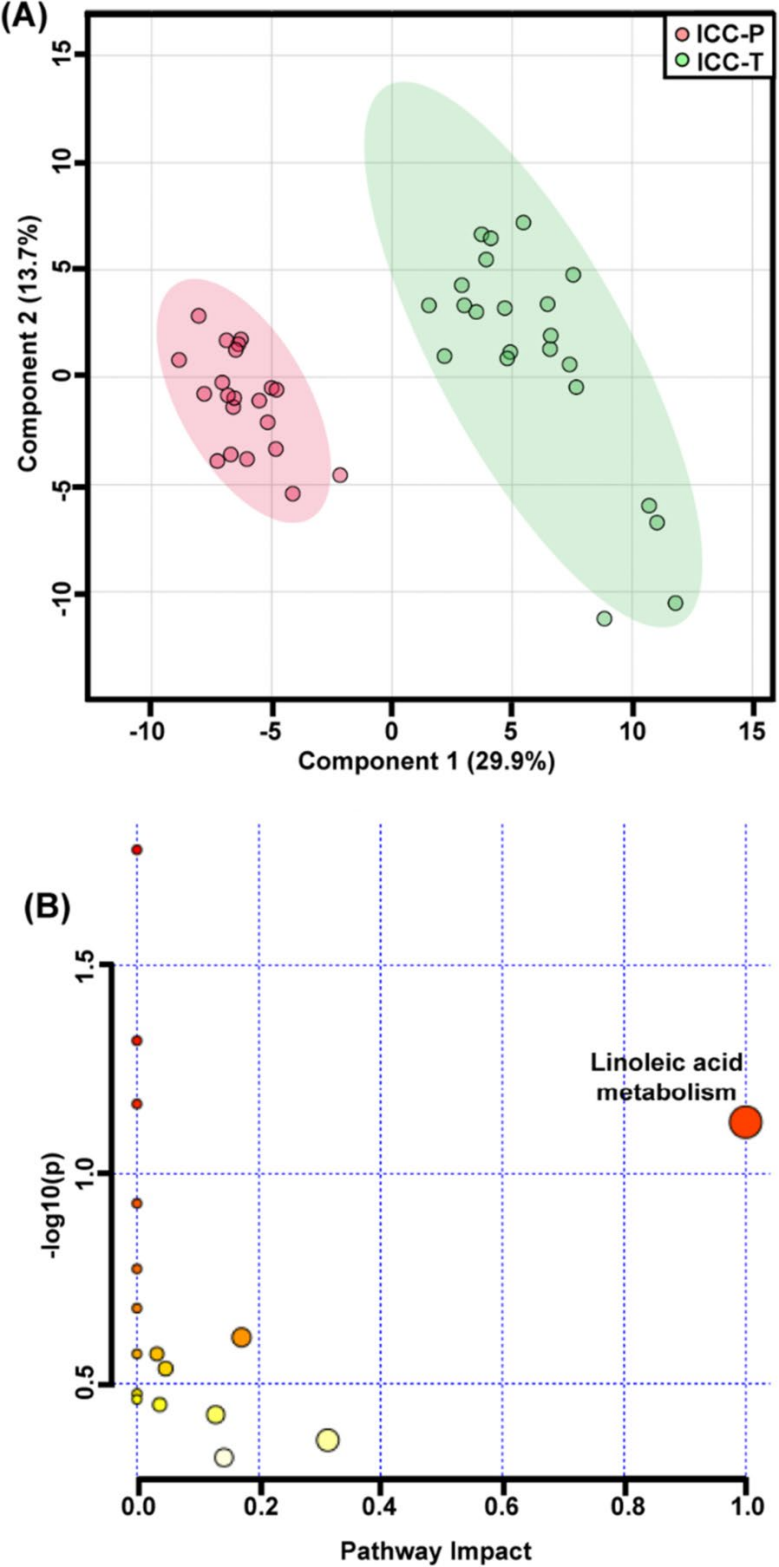
**A** PLS-DA of ICC carcinoma and para-carcinoma comparison. **B** Pathway analysis of differential metabolites between ICC-P and ICC-T

As shown in Fig. 3A, the ICC-T and ICC-P can be separated distinctly into two group, with R^2^X of 95% and Q^2^ of 91%. Then the variable importance for the projection (VIP) plot for the PLS-DA result was used to find out the significant contributed variates. 101 differential characteristics with VIP value greater than 1.0 were screened out to realize the distinction of ICC-T and ICC-P. Among them, 31 metabolites were successfully identified by comparing with METLIN database (Additional file 1: Table S1). Compared with the para-carcinoma tissues, there is a much higher level of myo-inositol and 2-propenoic acid in carcinoma tissues but lower levels of amino acids and carbohydrate metabolites, such as tryptiphan, D-allose, xylitol, D-ribonic acid and D-glucose, indicating a more vigorous energy metabolism in carcinoma tissues (Additional file 1: Fig. S2A).

**Fig. 4:**
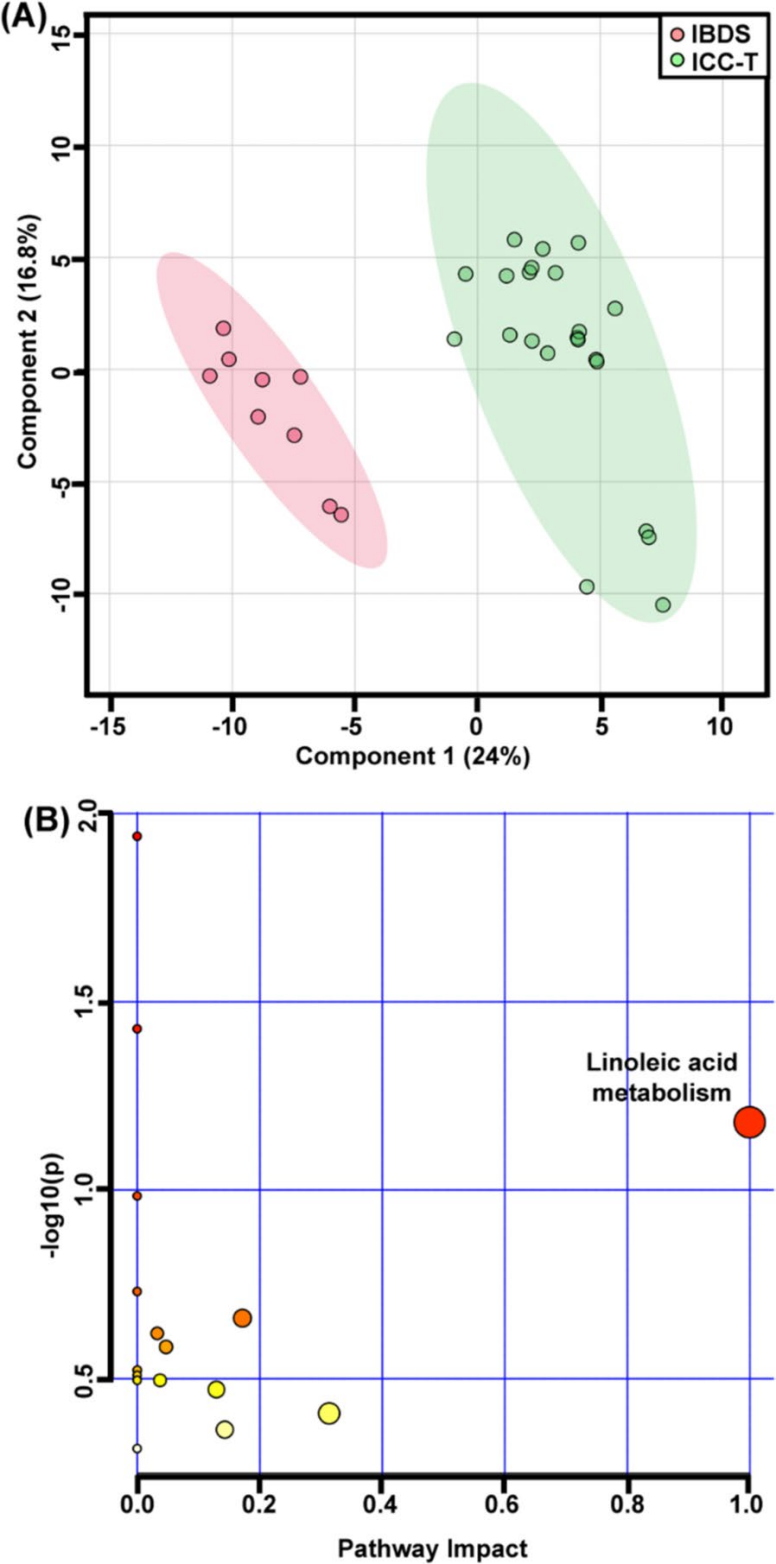
**A** PLS-DA of IBDS and ICC carcinoma comparison. **B** pathway analysis of differential metabolites between IBDS and ICC-T

To evaluate the accuracy of the above differential metabolites on distinguishing of ICC-P and ICC-T, receiver operating characteristic (ROC) analysis was performed using multivariate analysis mode. Area under the ROC curve (AUC) was used to express the accuracy of ROC analysis. When AUC value was close to 1.0, it indicates that the model has a high classification performance. Additional file 1: Table S2 shows the AUC values of these ICC-T differentially expressed metabolites, and all of them are greater than 0.5. Then, all these metabolites are used as classification features for ICC-P and ICC-T classification. The results show that the AUC value is higher than 0.99 (Additional file 1: Fig. S2B), indicating a great representativeness on distinguishing ICC-P and ICC-T (Fig. 4).

**Fig. 5:**
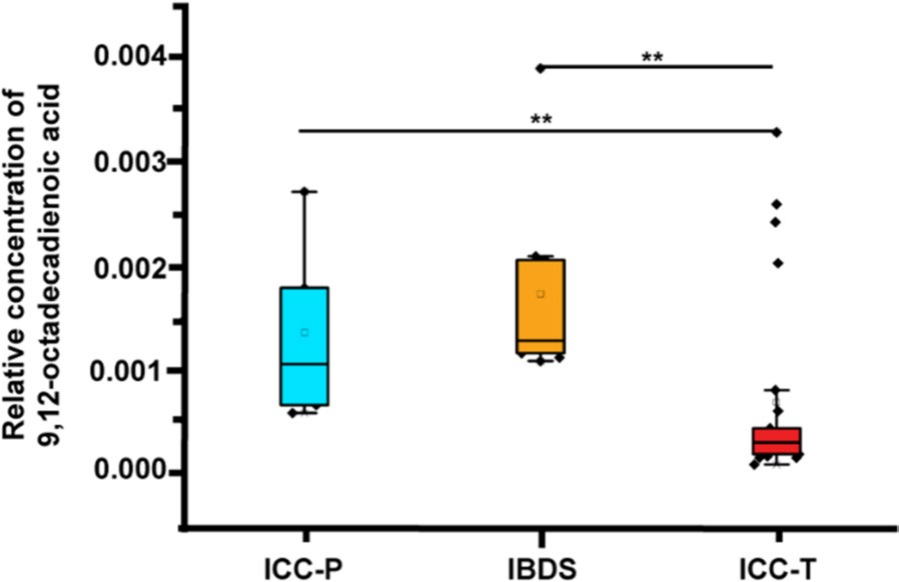
The relative concentration of 9,12-octadecadienoic acid among ICC-P, IBDS and ICC-T. **Indicate the *p* value < 0.01

KEGG-based pathway analysis was further preformed using above differential metabolites to demonstrate the potential metabolic function changes. Figure 3B shows the differential metabolic pathway between ICC-P and ICC-T, of which, the linoleic acid metabolism contributed to the biggest difference. In fact, the concentration of 9,12-octadecadienoic acid (also named as linoleic acid), the central metabolite of linoleic acid metabolite, was almost two times higher in ICC-P than in ICC-T (Fig. 5).

### Differences in metabolic fingerprints between ICC-T and IBDS

The PLS-DA result showed a clear separation of IBDS and ICC-T, with R2X and Q2 being 0.94 and 0.87, respectively (Fig. 4A). About 107 differential characteristics with VIP value greater than 1.0 were screened for the metabolic phenotypic distinguishing between IBDS and ICC-T. Among them, 28 metabolites were successfully identified (Additional file 1: Table S3). Comparing with IBDS tissues, 2-propenoic acid and myo-inositol were increased but other metabolites were significantly decreased in ICC-T samples (Additional file 1: Fig. S3A).

Univariate ROC analysis was carried out for evaluating the accuracy of these differential metabolites in distinguishing IBDS and ICC-T, and the resulted AUC values all higher than 0.5 (Additional file 1: Table S4). Next, these metabolites are used as classification features for multivariate ROC analysis and the results show that the AUC value is about 0.1 (Additional file 1: Fig. S3B), suggesting that these metabolites can well represent the metabolic differences between IBDS and ICC-T. The pathway analysis also demonstrated metabolic differences between IBDS and ICC-T were enriched in linoleic acid metabolism (Fig. 4B), and the concentration of 9,12-octadecadienoic acid in IBDS was almost two times higher than in ICC-T (Fig. 5).

### Inflammation and cholestasis promote the development of IBDS into ICC

**Fig. 6:**
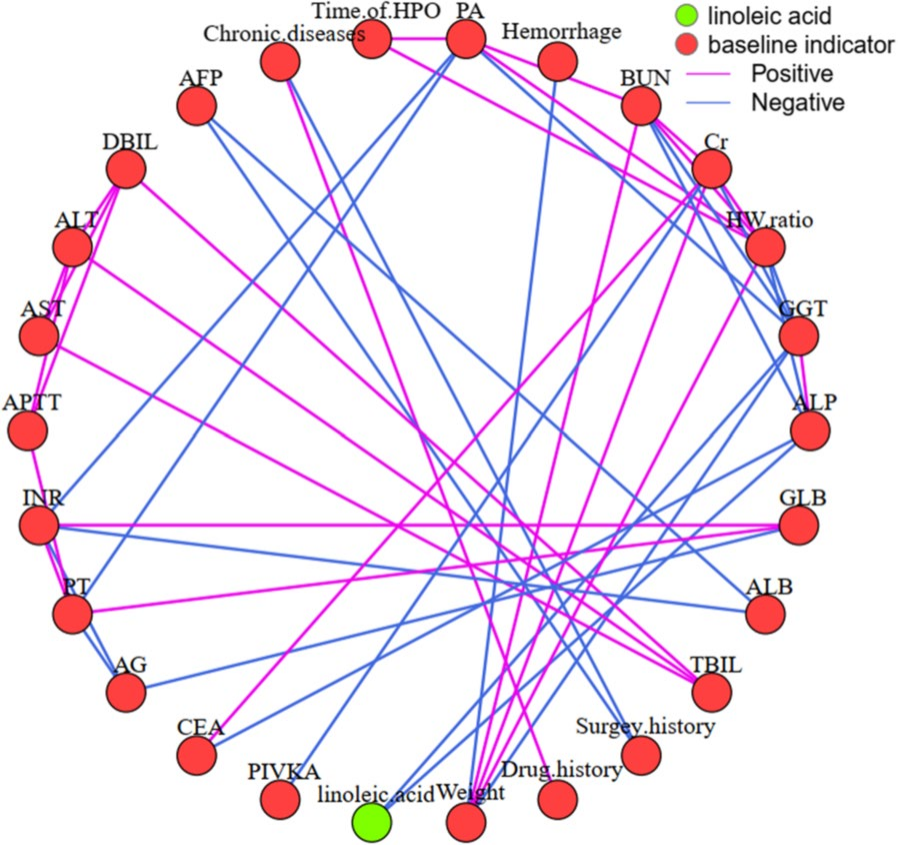
Spearman correlation network analysis of 9,12-octadecadienoic acid (linoleic acid) and baseline indicator. *AFP* alpha fetoprotein, *DBIL* direct bilirubin, *ALT* alanine aminotransferase, *AST* aspartate aminotransferase, *APTT* activated partial thromboplastin time, *INR* international normalized ratio, *PT* prothrombin time, *AG* albumin globulin ratio, *CEA* carcinoembryonic antigen, *PIVKA* protein induced by vitamin K absence or antagonist, *time of HPO* time of hepatic portal vein occlusion, *TBIL* total bilirubin, *ALB* albumin, *GLB* globulin, *ALP* alkaline phosphatase, *GGT* gamma-glutamyl transpeptidase, *HW ratio* height weight ratio, *Cr* creatinine, *BUN* blood urea nitrogen, *PA* prealbumin

9,12-Octadecadienoic acid, as an oxidizable metabolite, is sensitive to the oxidative pressure in the cellular microenvironment [22, 23]. Its abnormal decrease may be relevant with hepatic damage and/or carcinoma development. Prudently, individual indicators such as gender, age, medication history, and operation conditions etc. also may have contribution to 9,12-octadecadienoic acid decrease. Therefore, the Spearman correlation analysis between 9,12-octadecadienoic acid level and above indicators, including liver function indicators, tumor biomarkers, and individual indicators, was further performed. Intuitively, 9,12-octadecadienoic acid level of carcinoma tissue was negative correlation with gamma-glutamyl transpeptidase (GGT) and alkaline phosphatase (ALP) levels in peripheral blood, with 0.6 as threshold of correlation coefficient (Fig. 6). Meanwhile, levels of GGT and ALP have positive correlation between each other. Moreover, GGT level have negative correlation with other liver function biomarkers levels, such as prealbumin (PA), blood urea nitrogen (BUN) and creatinine (Cr). ALP level was also had negative correlation with BUN level. Taken together, the decrease of 9,12-octadecadienoic acid may be attribute the damage of liver function with increased biomarkers level of GGT and ALP as well as decreased biomarkers level of PA, BUN and Cr. It is important that the increase of GGT and ALP also are the characters of IBDS patients [24], which may contribute its evolution path to ICC.

## Discussion

Intrahepatic cholangiocarcinoma (ICC) is a type of hepatobiliary cancer located within the hepatic parenchyma, which histologically belong to the desmoplastic stroma-enriched adenocarcinoma with cholangiocyte differentiation and worse prognosis, and is the second most common primary hepatic malignant carcinoma [1–3]. In Asian, the incidence of ICC was about 0.5–1.0‰ [4–6, 19]. However, for population with IBDS, 2–10% patients eventually developed into ICC [19], almost forty times higher than normal population. Unfortunately, until recently, little is known about the carcinogenesis of IBDS. Therefore, further studying to elucidate potential mechanisms of IBDS evolution into ICC can provide effective IBDS monitoring biomarkers and symptomatic targets, which was essential to the treatment both on IBDS and ICC.

In this study, 34 participants including 25 ICC patients and 9 IBDS patients was recruited for metabolomic study to demonstrate potential metabolic phenotype differences between IBDS and ICC pathological tissues. Specifically, the IBDS tissue and para-carcinoma tissue have blurred metabolic phenotypic differences, but both of them essentially distinguished from carcinoma tissue of ICC. Therefore, we attempted to perform PLS-DA using ICC-T with IBDS and ICC-P, respectively, to find their potential differential metabolites and them-involved metabolic pathways. As shown in Figs. 3 and 4, metabolic differences between IBDS and ICC were enriched in linoleic acid metabolism (Fig. 4B), and the concentration of 9,12-octadecadienoic acid in IBDS was almost two times higher than in ICC (Fig. 5).

The mechanism of 9,12-octadecadienoic acid contributed to the development of IBDS is not clear. However, based on KEGG pathway retrospect, we noticed that 9,12-octadecadienoic acid is the main precursor for synthetic 13-S-hydroxyoctadecadienoic, a crucial regulator in down-regulating PPAR-δ to induce apoptosis in colorectal cancer cells [24]. Consequently, the abnormal level of 9,12-octadecadienoic acid indicates the decline of cancer suppressive ability of bile duct cells, and may lead to the evolution of IBDS to ICC under carcinogen induction.

The characteristic of IBDS including recurrent inflammation and cholestasis may be contribute to the disturbance of linoleic acid metabolic pathway. In the period of IBDS inflammation, the concentration of NF-kB usually increases significantly, which can stimulate the secretion of growth factors and lead to the cell cancerization [25]. In normal cells, the operation of linoleic acid metabolic pathway can ensure the normal synthesis of 13-S-hydroxyoctadecadienoic, thus promoting the function of PPAR-δ [26]. Importantly, one of functions of PPAR-δ is to regulate NF-kB signal to protect the liver [26]. Therefore, when linoleic acid metabolism is inhibited, this liver protective effect is weakened, which may accelerate the occurrence of cancer.

The normal secretion of bile acids is an important guarantee to maintain the absorption of fatty acids, which will be hindered in IBDS patients and consequently results in the decrease of bile acids level in intestinal, eventually affects the normal absorption of fatty acids. An animal experiment showed that the content of linoleic acid in the plasma of mice with bile shunt was significantly reduced [27]. Taken together, the cholestasis of IBDS may affect the absorption of linoleic acid, which will weaken the anti-inflammatory effect of 13-S-hydroxyoctadecadienoic, and finally lead to the accelerated carcinogenesis of hepatobiliary cells induced by carcinogens.

In general, the cancerous process usually takes more than a decade. During this period, it is difficult to obtain persistent linoleic acid metabolism abnormal without causing perturbation of relevant liver function or tumor biomarkers. Therefore, the correlation between 9,12-octadecadienoic acid level and indicators including liver function indicators, tumor biomarkers, and individual indicators was analyzed. Intuitively, 9,12-octadecadienoic acid level of pathological tissue was negative correlation with GGT and ALP level in peripheral blood, with 0.6 as threshold of correlation coefficient (Fig. 6). Meanwhile, GGT have negative correlation with other liver function biomarkers, such as PA, BUN and Cr. ALP was also had negative correlation with BUN level. As a consequence, the decrease of 9,12-octadecadienoic acid level in ICC pathological tissues may relevant to the increase of GGT and ALP level, as well as the decrease of liver function biomarkers of PA, BUN and Cr. This character of biomarkers always indicates a damage of liver function. Mechanistically, the 9,12-octadecadienoic acid can modulate the hepatic immune microenvironment in the hepatitis mouse models [27], stimulate the expression of neutrophils and macrophages [27, 28], up-regulate the level of inflammatory factors [29], induce high expression of carnitine palmitoyltransferase leading to increased apoptosis of CD4^+^ T cells [30], and ultimately contribution to the HCC progression [31].

Like traditional liver cancer markers alpha-fetoprotein and CA19-9 [32, 33], 9,12-octadecadienoic acid associated liver function indicators, GGT and ALP, have also been found to be related to the occurrence and progression of liver cancer. Most recently, Li et al. reported that the GGT/ALP ratio combined with GGT/ aspartate aminotransferase (AST) ratio and alanine aminotransferase (ALT)/AST ratio can be used for accurate screening alpha fetoprotein (AFP)-negative hepatocellular carcinoma (HCC) [34], confirming the important role of GGT and ALP in liver tumor evolution. In fact, IBDS patients always companied with increased GGT and ALP level [26], which may contribute its evolution path to ICC. GGT, as a biomarker of cellular proliferation, was previously found increase in carcinogens induced HCC rat models [35]. Moreover, high level of GGT in carcinoma tissues can increase the GSH transmembrane uptake, which can further active GSH-dependent chemoresistance [36], suggesting a worse prognosis. On the other hand, ALP as a marker of terminal differentiation can be involve into multiply cell activities, such as differentiation and cycle regulation [37–39]. Its abnormal increase previously be reported as an indicator for hepatocarcinogenesis and prognosis [40, 41]. Consequently, long-term monitoring of metabolites from linoleic acid metabolism pathway and protein indicators of liver function in IBDS patients has important guiding significance for the monitoring of IBDS carcinogenesis. More importantly, it is necessary to further clarify the causal relationship between linoleic acid pathway disturbance and changes in liver function, which can provide an important therapeutic target for both IBDS and ICC.

## Conclusions

In this study, we explored the metabolic phenotype difference between IBDS and ICC. Although, the IBDS tissue and para-carcinoma tissue have blurred metabolic phenotypic differences, both of them essentially distinguished from carcinoma tissue of ICC. Metabolic differences between IBDS and ICC were enriched in linoleic acid metabolism pathway, and the concentration of 9,12-octadecadienoic acid in IBDS was almost two times higher than in ICC. The correlation between 9,12-octadecadienoic acid level and baseline information of patients demonstrated that 9,12-octadecadienoic acid level of pathological tissue was negative correlation with GGT and ALP level in peripheral blood. These two indicators were all cancerization marker for hepatic carcinoma and disease characteristic of IBDS. This study provides valuable metabolic clues for the carcinogenesis mechanism of IBDS, and has guidance significance for long-term monitoring and symptomatic treatment of IBDS.

## Supplementary Information

**Additional file 1.** Experimental details and metabolomics results.

## Data Availability

All data generated or analyzed during this study are included in this published article and its supplementary information files. Protocols and raw data are available at MetaboLights database (www.ebi.ac.uk/metabolights/MTBLS3646). Public access to the MetaboLights database is open.
