# Supplementary material for: Linoleic acid pathway disturbance contributing to potential cancerization of intrahepatic bile duct stones into intrahepatic cholangiocarcinoma
Source: BMC Gastroenterol. 2022 May 30;22:269. doi: 10.1186/s12876-022-02354-2 (PMC9153149; doi:10.1186/s12876-022-02354-2)
Supplement: Supplementary file 1 — Additional file 1. Experimental details and metabolomics results. [file 12876_2022_2354_MOESM1_ESM.docx]

Linoleic acid pathway disturbance contributing to potential cancerization of intrahepatic bile duct stones into intrahepatic cholangiocarcinoma

Jun Li,^1, #^ Jiongjiong Lu,^2, #^ Shaodong Lv,^3, #^ Shujun Sun,^4^ Caifeng Liu,^1^ Feng Xu,^1^ Haiying Sun,^1^ Jiamei Yang,^2^ Xinjun Wang,^5, 6 *^ Xingyang Zhong^1, *^ and Junhua Lu^3, *^

^1 The 1st Department of Hepatic Surgery, Eastern Hepatobiliary Surgery Hospital, Second Military Medical University, Shanghai, China.^

^2 Department of Special Treatment, Eastern Hepatobiliary Surgery Hospital, Second Military Medical University, Shanghai, China.^

^3 The 5st Department of Hepatic Surgery, Eastern Hepatobiliary Surgery Hospital, Second Military Medical University, Shanghai, China.^

^4 School of biology and food engineering, Fuyang Normal University, Fuyang, China.^

^5 Translational Medical Center for Stem Cell Therapy and Institute for Regenerative Medicine, Shanghai East Hospital, Shanghai Key Laboratory of Signal-ing and Disease Research, School of Life Sciences and Technology, Tongji University, Shanghai, China.^

^6 Shanghai Institution of Gut Microbiota Research and Engineering Development, Tenth People’s Hospital of Tongji University, Tongji University School of Medicine, Shanghai, China.^

^# Co-first authors^ ^with equal contribution.^

^* Corresponding authors with equal contribution. E-mail of Xinjun Wang:^ [^xjwang16@fudan.edu.cn^](mailto:xjwang16@fudan.edu.cn)^, Xingyang Zhong:^ [^13585117406@163.com^](mailto:13585117406@163.com)^, Junhua Lu: lujunhua888888@sina.com.^

**Gas Chromatography-Mass Spectrometry analysis (GC-MS).** Tissue samples were stored at -80℃ until GC-MS assay. 50 mg tissues were homogenized using 150 μL normal saline. Each 100 μL mixture was mixed with 250 μL acetonitrile. Then, the mixture was placed into a screw tube, vortex-mixed for 3min, ultrasound in ice bath for 10 min and then centrifugated for separating supernatant (1000r/min, 10min). Supernatant was transferred into a GC vial and evaporated to dryness under N2 blowing at 30℃. 50 μL of methoxyamine in pyridine (15 g/L) was added to the GC vial, vortex-mixed for 1 min, and the methoximation reaction was carried out for 60 min rocking in a shaker at 70℃, then 50 μL of BSTFA plus 1% TMCS was added to the samples for trimethylsilylation for another 1 h at 70∘C. At last, 150 μL of heptane containing external standard methyl myristate was added to the GC vial, and the solution was centrifugated to separate supernatant for GC-MS based metabolomics analysis.

**Metabolite identification.** As to the profiles obtained from GC-MS, wispy shifts in retention time between fingerprints occur due to experimental variations and column aging. When the total ion current chromatograms (TICs) were obtained, peak-alignment or warping techniques are commonly applied to compensate for minor shifts in retention times. Thus, in the subsequently data processing, the same variable manifested synchronous information in every profile. For this purpose, all the GC-MS raw flies after being converted to CDF format via the software coming with Agilent MSD workstation, were subsequently processed by the XCMS toolbox (<http://metlin.scripps.edu/download/>) using XCMS’s default settings with the following exceptions: xcmsSet (full width at half maximum: fwhm = 5; S/N cutoﬀ value: snthresh = 10, max = 15), group (bw = 5). The resulting table (CSV file) was exported into Microsoft Excel (Microsoft Inc., USA), where normalization was performed prior to multivariate analyses. Those metabolites were identified by searching in NIST 2005 database and verified by standards.

**Statistical analysis.** Data were analyzed by parametric and nonparametric statistical tests using SPSS (version 19.0) and Simca-P (version 11.5). Continuous data were compared by t-test. Differences in metabolic profiles on GC/MS were determined by principal component analysis (PCA) and partial least squares discriminant analysis (PLS-DA). To validate the importance of the metabolites, and to further gauge their ability to distinguish from ICC to IBDS and controls, their potential predictive utility for ICC was assessed by receiver operating characteristic (ROC) curve analysis. ROC analysis was performed using MS peak areas corresponding to the metabolite concentrations in each group. Areas under the ROC curve were calculated using the ROCR package (classifier visualization in R). Pathways involved by metabolites were analyzed with Kyoto Encyclopedia of Genes and Genomes (KEGG) (http://www.genome.ad.jp/kegg/) and Metabolites Biological Role (MBRole) (http://csbg.cnb.csic.es/mbrole) were based to select the related pathway. Many references were searched to give the biochemical interpretation of changed metabolites disturbed pathways of ICC.


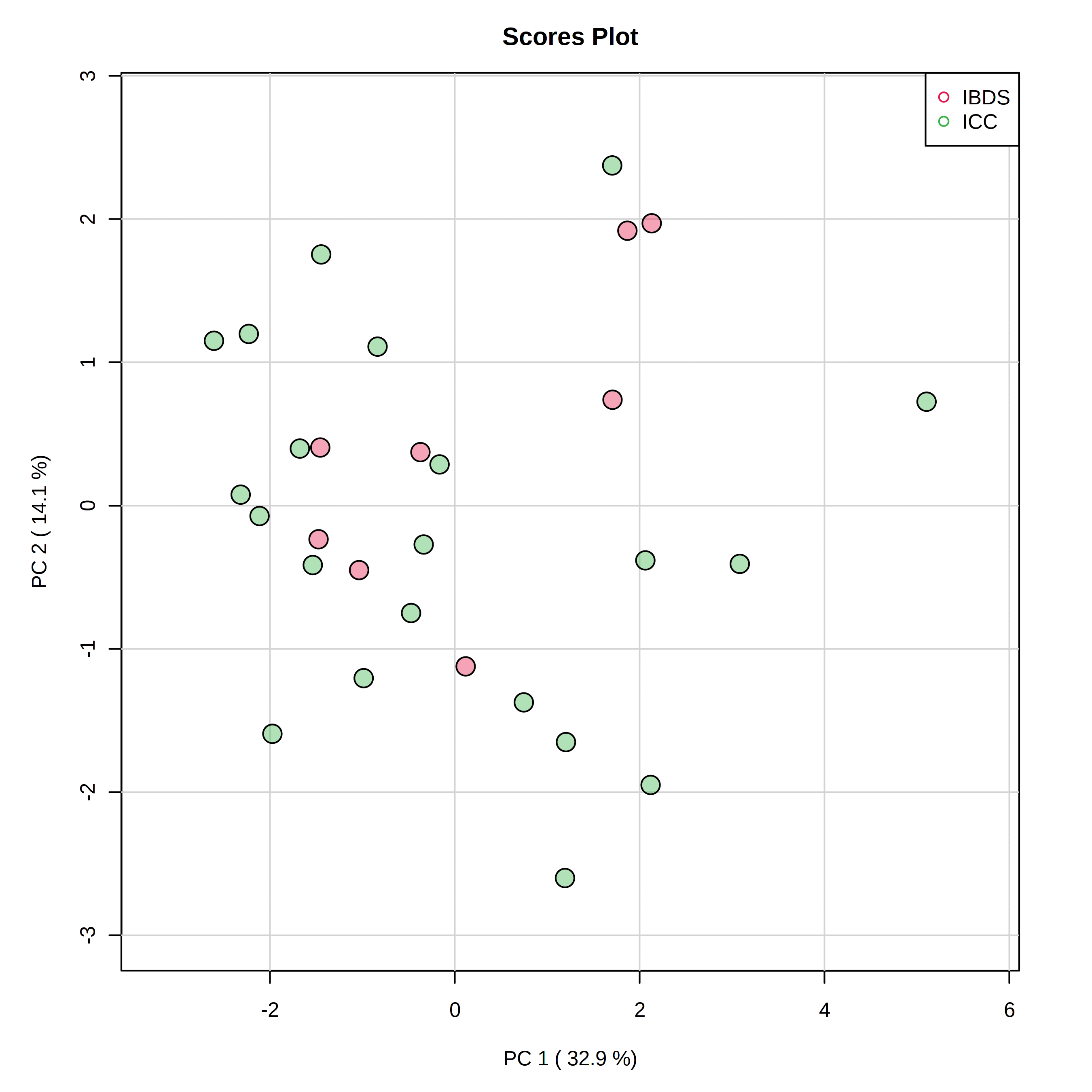


**Figure S1.** PCA score plot of clinical baseline information between IBDS and ICC patients.


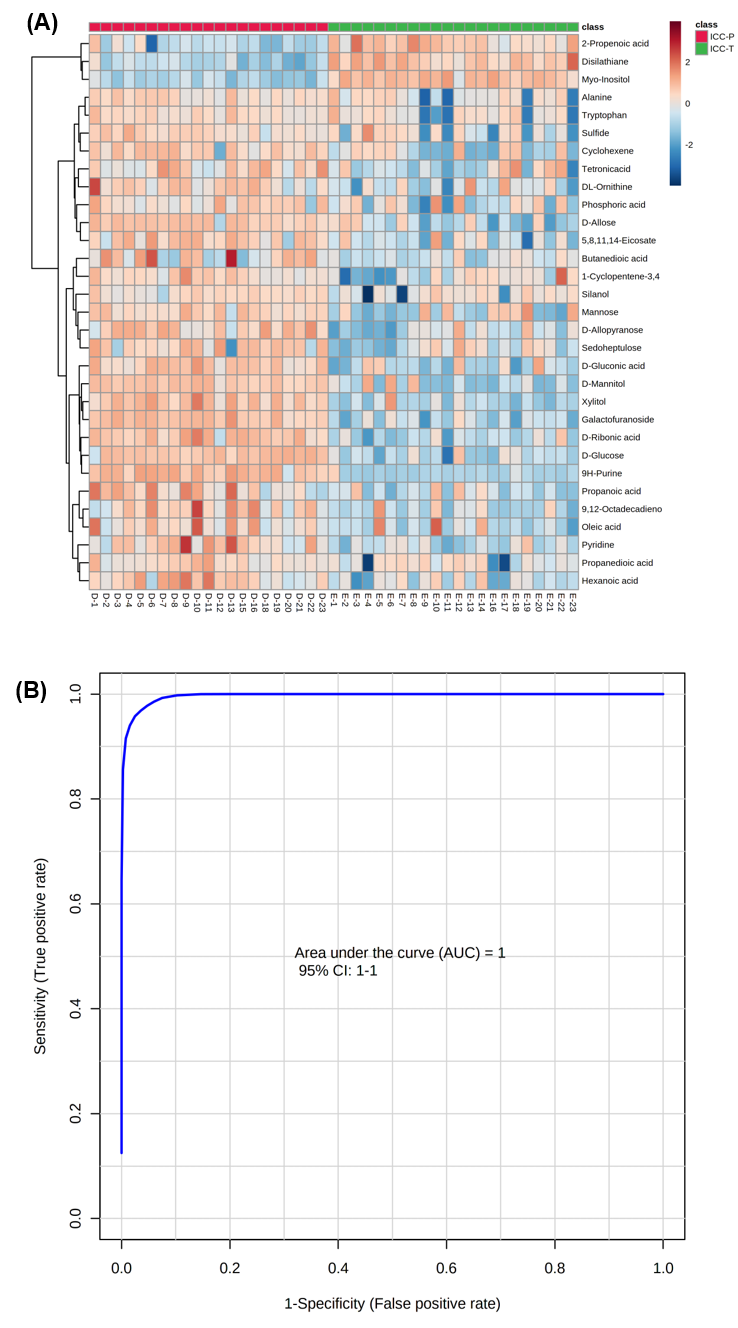


**Figure S2.** (A) Heatmap analysis of ICC associated metabolites among pathological tissues and para-cancer tissues. (B) ROC curve of distinguishing ICC pathological tissues and para-cancer tissues using ICC differentially expressed metabolites.


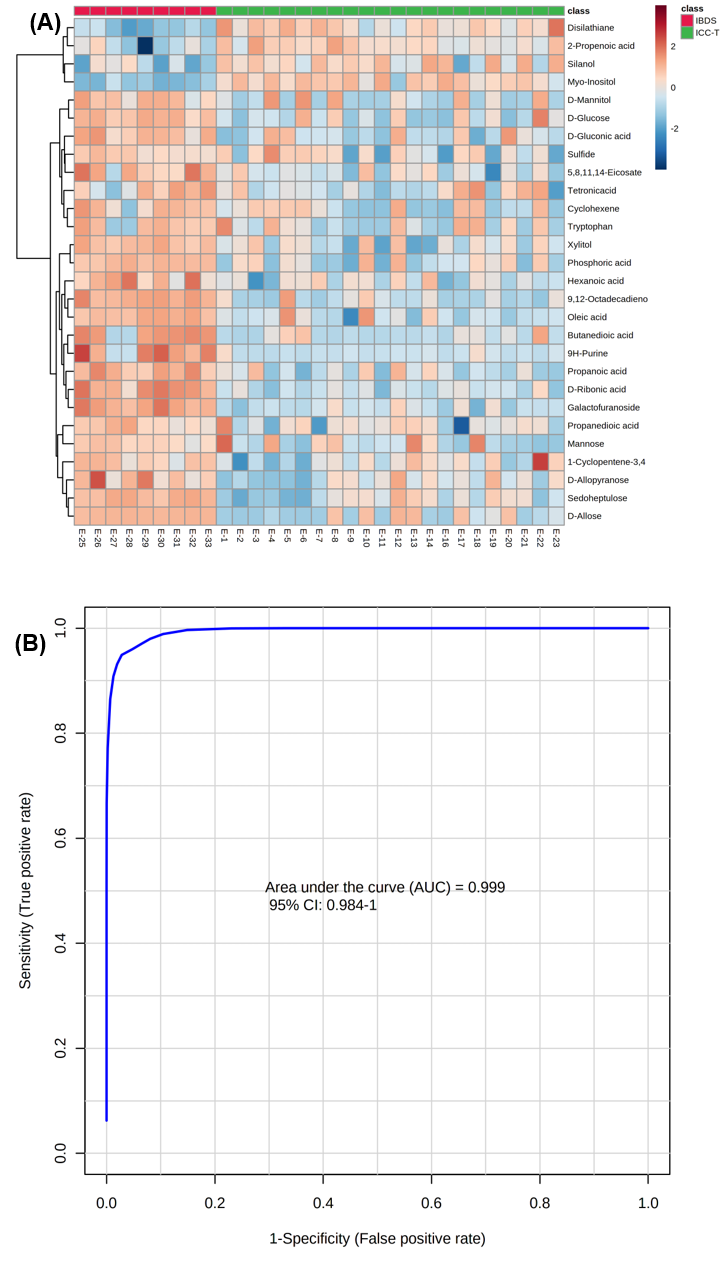


**Figure S3.** (A) Heatmap analysis of ICC associated metabolites among pathological tissues and IBDS tissues. (B) ROC curve of distinguishing ICC pathological tissues and IBDS tissues using ICC differentially expressed metabolites.

**Table S1.** 31 ICC pathological tissues differentially changed metabolites relative to ICC para-cancer tissues.

| Metabolic characteristic | Metabolite | VIP of PLS-DA |
| --- | --- | --- |
| M353T1509 | 9H-Purine | 2.6177 |
| M331T1402 | D-Mannitol | 2.5396 |
| M202T1585 | D-Allose | 2.3512 |
| M361T1423 | Galactofuranoside | 2.1928 |
| M204T1456 | D-Glucose | 1.9669 |
| M333T1432 | D-Gluconic acid | 1.841 |
| M198T1027 | Cyclohexene | 1.6793 |
| M217T1118 | Xylitol | 1.5818 |
| M73T1091 | D-Ribonic acid | 1.4855 |
| M41T1352 | Mannose | 1.3767 |
| M220T978 | Tetronicacid | 1.3668 |
| M333T1480 | 1-Cyclopentene-3,4-dione | 1.3604 |
| M191T1333 | D-Allopyranose | 1.3384 |
| M91T2054 | 5,8,11,14-Eicosatetraenoic acid | 1.3297 |
| M88T798 | Sulfide | 1.3191 |
| M122T1752 | 9,12-Octadecadienoic acid | 1.2743 |
| M202T1735 | Tryptophan | 1.2565 |
| M292T733 | Propanoic acid | 1.2394 |
| M130T488 | Pyridine | 1.228 |
| M73T1599 | Myo-Inositol | 1.2279 |
| M248T853 | Hexanoic acid | 1.189 |
| M155T977 | Alanine | 1.1395 |
| M161T1367 | Sedoheptulose | 1.1376 |
| M142T1233 | DL-Ornithine | 1.1287 |
| M171T318 | Disilathiane | 1.0922 |
| M247T713 | Butanedioic acid | 1.0862 |
| M84T1758 | Oleic acid | 1.0861 |
| M216T524 | Propanedioic acid | 1.0707 |
| M129T652 | Silanol | 1.0537 |
| M361T1173 | Phosphoric acid | 1.042 |
| M55T411 | 2-Propenoic acid | 1.0347 |

**Table S2.** AUC value of 31 ICC pathological tissues differentially changed metabolites relative to ICC para-cancer tissues.

| Metabolite | AUC |
| --- | --- |
| Galactofuranoside | 0.99784 |
| Myo-Inositol | 0.99784 |
| Disilathiane | 0.99351 |
| 9H-Purine | 0.99134 |
| D-Glucose | 0.96753 |
| D-Ribonic acid | 0.96104 |
| Xylitol | 0.95671 |
| 2-Propenoic acid | 0.94156 |
| D-Allose | 0.91126 |
| D-Allopyranose | 0.88961 |
| Pyridine | 0.88312 |
| D-Gluconic acid | 0.87229 |
| D-Mannitol | 0.86797 |
| 9,12-Octadecadienoic acid | 0.86147 |
| Oleic acid | 0.84632 |
| 5,8,11,14-Eicosatetraenoic acid | 0.84632 |
| Hexanoic acid | 0.83117 |
| Propanoic acid | 0.82035 |
| DL-Ornithine | 0.80087 |
| Sedoheptulose | 0.80087 |
| Cyclohexene | 0.7619 |
| 1-Cyclopentene-3,4-dione | 0.75974 |
| Tetronicacid | 0.75108 |
| Silanol | 0.72511 |
| Sulfide | 0.71212 |
| Alanine | 0.71212 |
| Mannose | 0.71212 |
| Butanedioic acid | 0.69913 |
| Propanedioic acid | 0.69697 |
| Tryptophan | 0.69264 |
| Phosphoric acid | 0.68615 |

**Table S3.** 28 ICC pathological tissues differentially changed metabolites relative to IBDS tissues.

| Metabolic characteristic | Metabolite | VIP of PLS-DA |
| --- | --- | --- |
| M307T1372 | D-Allose | 3.1184 |
| M217T1424 | Galactofuranoside | 2.5098 |
| M293T1458 | D-Glucose | 2.4318 |
| M354T1508 | 9H-Purine | 2.3371 |
| M311T1115 | Xylitol | 2.0805 |
| M179T923 | Butanedioic acid | 2.0597 |
| M80T1753 | 9,12-Octadecadienoic acid | 1.9861 |
| M89T1758 | Oleic acid | 1.9433 |
| M345T1397 | D-Mannitol | 1.9209 |
| M307T1091 | D-Ribonic acid | 1.9153 |
| M333T1432 | D-Gluconic acid | 1.6577 |
| M192T648 | Silanol | 1.6477 |
| M357T1174 | Phosphoric acid | 1.5548 |
| M91T2054 | 5,8,11,14-Eicosatetraenoic acid | 1.4667 |
| M160T1369 | Sedoheptulose | 1.4574 |
| M198T1027 | Cyclohexene | 1.4289 |
| M189T733 | Propanoic acid | 1.3787 |
| M191T1333 | D-Allopyranose | 1.2593 |
| M334T1481 | 1-Cyclopentene-3,4-dione | 1.2425 |
| M171T318 | Disilathiane | 1.2354 |
| M203T1734 | Tryptophan | 1.2177 |
| M55T411 | 2-Propenoic acid | 1.2068 |
| M175T1354 | Mannose | 1.1901 |
| M248T853 | Hexanoic acid | 1.1813 |
| M88T798 | Sulfide | 1.1574 |
| M73T1599 | Myo-Inositol | 1.1177 |
| M188T520 | Propanedioic acid | 1.0664 |
| M220T978 | Tetronicacid | 1.0505 |

**Table S4.** AUC value of 28 ICC pathological tissues differentially changed metabolites relative to IBDS tissues.

| Metabolite | AUC |
| --- | --- |
| Galactofuranoside | 1 |
| D-Ribonic acid | 0.99495 |
| Disilathiane | 0.98485 |
| Myo-Inositol | 0.98485 |
| Sedoheptulose | 0.97475 |
| 9,12-Octadecadienoic acid | 0.96465 |
| D-Allose | 0.9596 |
| Propanoic acid | 0.94444 |
| Xylitol | 0.93939 |
| Phosphoric acid | 0.91919 |
| Oleic acid | 0.90909 |
| D-Glucose | 0.89899 |
| Hexanoic acid | 0.88384 |
| 5,8,11,14-Eicosatetraenoic acid | 0.88384 |
| 9H-Purine | 0.87374 |
| 2-Propenoic acid | 0.86869 |
| D-Gluconic acid | 0.85859 |
| D-Allopyranose | 0.85354 |
| Butanedioic acid | 0.83333 |
| Propanedioic acid | 0.82323 |
| 1-Cyclopentene-3,4-dione | 0.82323 |
| Silanol | 0.81818 |
| D-Mannitol | 0.77273 |
| Cyclohexene | 0.76768 |
| Mannose | 0.73737 |
| Sulfide | 0.71212 |
| Tetronicacid | 0.69697 |
| Tryptophan | 0.66162 |
| Galactofuranoside | 1 |
| D-Ribonic acid | 0.99495 |
| Disilathiane | 0.98485 |
